# Effects of Sleep Duration on Falls in a West Virginia Population-Based Study, BRFSS, 2018

**DOI:** 10.13023/jah.0302.03

**Published:** 2021-05-03

**Authors:** R. Constance Wiener, Christopher Waters

**Affiliations:** Dental Practice and Rural Health, West Virginia University; Department of Dental Research, West Virginia University

**Keywords:** Appalachia, BRFSS, fall, injury, West Virginia, sleep

## Abstract

**Introduction:**

West Virginia is a state in which most counties are rural, as well as a state with multiple health disparities among its population. The purpose of this study was to determine the association of sleep duration and falls for non-institutionalized West Virginia adults, aged 40 years and above, using the National Sleep Foundation’s definition of “may be appropriate” and “not recommended” sleep durations for specific ages.

**Methods:**

Behavior Risk Factor Surveillance System (BRFSS) 2018 data concerning West Virginia residents were extracted for sleep duration and number of falls within the previous year. Data were analyzed with Chi square and logistic regression analyses on falls.

**Results:**

There were 2780 participants, aged 45 years and above. Slightly more than half (51.0%) were female. In adjusted logistic regression analysis, the adjusted odds ratio for falls in participants who did not have the recommended sleep duration was 1.77; 95%CI: 1.38, 2.27; *p*<0.0001 as compared with participants who did have the recommended sleep duration.

**Conclusion:**

Inadequate sleep duration, based on age, was associated with ≥1 falls within the previous year in a West Virginia Appalachian population.

## INTRODUCTION

Fall injuries are public health challenges and a leading cause of functional impairment. Fall injuries often result in fractures, pain, physical limitations, dependency, and premature death.^1^,^2^ The Centers for Disease Control and Prevention (CDC) reports that one fourth of older adults fall annually, and fewer than half of the people who fall report the fall to his or her physician.^3^ Nearly 20% of falls result in serious injuries, such as fractures and head injuries. Annually, there are three million fall-related emergency department visits. Medical costs for falls in 2015 approached $50 billion; and, from 2007 to 2016, the death rate from falls increased 30%.^3^ Relevant factors that contribute to falls are uneven surfaces, comorbidities, musculoskeletal deficits, medications known as “FRIDS” (fall-risk increasing drugs), vision issues, balance/gait, foot pain, inappropriate shoes, tripping hazards, cognitive impairment, diminished/poor reaction time, lack of exercise,^1^, ^4^–^7^ and potentially the lack of vitamin D^8^–^10^ and the need for creatinine-rich foods/supplements.^11^,^12^

Many of these factors are associated with sleep disorders. Researchers indicated that poor sleep quality, insomnia, sleep disturbances, ≤5 hours of sleep, and ≥10 hours of sleep were risk factors for recurrent falls in women, aged 50–79 years.^13^ Researchers found an association of napping/short sleep duration and falls in women^7^,^14^ and daytime sleepiness and falls in men.^15^ In a study of older adults in self-care and assisted-care villages in New South Wales, researchers reported that participants who slept <6 hours at night or napped >30 minutes during the day were three times as likely to have multiple falls in the follow-up year.^16^

There is a plausible biological mechanism linking sleep disorders and increased risk of falls. Sleep is necessary to upregulate functions such as growth, repair, immunologic functions, and the consolidation of neural input,^17^ bone turnover, and muscle strength.^13^ Adults frequently have difficulty with sleep latency, sleep duration, daytime sleepiness, frequently awakening, and poor-quality sleep. Poor sleep may lead to decreased muscle strength, cognitive impairment, depression,^13^ and poor balance,^18^ all of which can increase the risk for falls.

Researchers have indicated that throughout the world, there are regional differences in sleep quality and insomnia. For example, in terms of rural/urban regions, in one study in China, rural residents had more sleep disorders than urban residents.^19^ In a study in India, rural residents reported better sleep quality than urban residents.^20^ In a nationwide U.S. study of participants, aged ≥18 years, rural/urban residence did not have a significant relationship with enough sleep.^21^

West Virginia is a state in which most counties are rural.^22^ It is a state that does not have any cities with a population of 50,000.^22^ It is also a state in which there are multiple health disparities.^23^ To the knowledge of the authors, the influence of sleep disorders on falls has not been examined in this rural Appalachian population. Additionally, previous researchers who have studied the relationship of sleep and falls have examined older adults and have used sleep times of ≤5 hours or ≥10 hours as cut points for their research. There is a lack of studies including adults aged ≥45 years. There is also a lack of studies using the National Sleep Foundation’s definitions of “may be appropriate sleep,” and “not recommended” amount of sleep.^24^ The purpose of this study was to determine the association of sleep and falls for non-institutionalized West Virginia adults, aged ≥45 years, using the National Sleep Foundation’s definition of “may be appropriate” and “not recommended” sleep durations for specific ages. The null hypothesis for this study was that there is no difference in falls between participants who had appropriate hours of sleep and participants who did not have the recommended hours of sleep.

## METHODS

### Ethics Statement

This study received West Virginia University IRB acknowledgement of nonhuman subject research (Protocol number 2002910535).

### Study Design

The study has a cross-sectional observational study design. The STROBE (Strengthening the Reporting of Observational Studies in Epidemiology) reporting guidelines were followed.^25^

### Data Source

Data were retrieved from the Centers for Disease Control and Prevention’s Behavioral Risk Factor Surveillance System, (BRFSS) 2018, available at https://www.cdc.gov/brfss/annual_data/annual_2018.html. BRFSS is a system of telephone surveys conducted by states under the Centers for Disease Control and Prevention for public health surveillance data. Interviewers contact U.S. residents and request responses to interview questions concerning health-related risk behaviors.^26^ Aggregated state BRFSS surveys include more than 400,000 adult interviews annually.^26^ Data approximate the nation if appropriate considerations are made for the complex survey design, including survey weights, stratification, and primary sampling unit as indicated.

### Sample

The sample for this study were participants from West Virginia who were aged 45 years and above with complete data on falls, sleep duration, and the other covariates.

### Key Dependent Variable

The key variable for this study was falls. Participants were asked “In the past 12 months, how many times have you fallen?”^26^ In the raw national data, 26.81% of participants reported having fallen at least one time during the previous 12 months. Data for this variable were dichotomized into yes (at least one fall during the previous 12 months) and no (no falls during the previous 12 months).

### Key Independent Variable

The key independent variable was sleep duration for specific age. Participants were asked “On average, how many hours of sleep do you get in a 24-hour period?”^26^ The responses were dichotomized into “not recommended for their age,” and “may be appropriate for their age,” based on the National Sleep Foundation sleep times. For individuals aged 26–64 years, a not recommended sleep duration is <6 hours and >10 hours; and for individuals ≥65 years, a not recommended sleep duration is <6 hours and >9 hours.^24^ Therefore, “may be appropriate” sleep durations in this study were 6 to 10 hours for individuals aged 26–64 years; and 6–9 hours for individuals ≥65 years.^24^

### Covariates

Other variables included in the study were: gender (male, female); age (45–54 years, 55–64 years, 65 and above); race (white, nonwhite); education (less than high school graduate, high school graduate, some college or above); health insurance (yes, no); body mass index (BMI) (under/normal, defined as a BMI<25; overweight, defined as a BMI of 25 to <30; obese, defined as a BMI>30); exercise (yes, no); chronic disease (positive response to having arthritis, cardiovascular disease, depression, chronic obstructive lung disease, or diabetes, no); and sun exposure of at least 30 minutes in a week (yes, no). Sun exposure was determined with self-reported responses to two BRFSS questions: “On weekdays, in the summer, how long are you outside between 10 a.m. and 4 p.m.?” and “On weekends, in the summer, how long are you outside between 10 a.m. and 4 p.m.?”^26^ If the response to both questions was “less than half of an hour,” sun exposure was coded as “no.” Otherwise, it was coded as “yes” unless the participant reported uncertainty, refusal to respond, or if the question was not asked, in which case the participant was not included in the study.

### Statistical Analysis

The sample was described with weighted percentages and frequency. Chi-square tests were completed to determine bivariate associations of falls with the variables of interest. Unadjusted and adjusted logistic regression analyses were conducted on falls. Study design, weights, and state selection (eligible population) were taken into account in the analyses. SAS® version 9.4 (SAS Institute, Inc., Cary NC) was used for the analyses. The significance level was set, a priori, at p<0.05.

## RESULTS

The sample included 2780 participants, 51.0% of whom were female. There were 40.7% who were aged ≥65 years. As is representative of the West Virginia population at large, there were 94.2% who were white, 68.2% who were high school graduates. The prevalence of falls was 31.1%. Details of the sample description are in Table 1.

In Chi-square bivariate analyses, there was a significant association of falls with not having recommended sleep. Additionally, less education, older age, higher BMI, not exercising, lack of sun exposure, and chronic disease were significantly associated with higher fall prevalence. Details are presented in Table 2.

In unadjusted logistic regression on falls, not recommended sleep duration was significantly associated with falls as compared with appropriate sleep duration (Odds Ratio=2.26; 95%CI: 1.77, 2.82; p<0.0001). The relationship was attenuated, but significant in adjusted analyses (Adjusted Odds Ratio [AOR] = 1.77; 95%CI: 1.38, 2.27; p=<0.000). Results are presented in Table 3.

## DISCUSSION

In this study of West Virginia residents, aged 45 years and above, not having recommended durations of sleep for their age was significantly associated with ≥1 falls within the previous year. The relationship was strong and remained so in adjusted analyses. These results support previous research limited to older adults,^16^ only older females,^13^ and only older males.^15^ This research extends the literature as the researchers used recommended durations of sleep based on the National Sleep Foundation’s definitions. There were 8,173,139 unintentional falls resulting in nonfatal emergency department visits in the U.S. in 2018.^22^ There were 38,707 fall deaths in the U.S. in 2018.^27^ In this analysis of West Virginia residents, it was found that the prevalence of falls differs by age.

Falls are the result of many factors, such as the factors that are extrinsic to the individual (slippery floors, poor lighting, footwear, incorrect use of assistive device); intrinsic to the individual (strength, balance, sleep disturbance, comorbidities, types of medications, age); and bio–psycho–social–ecologic–environmental (marital status, education level, income, place of residence). Previous researchers have suggested that most falls are likely from a combination of extrinsic and intrinsic factors.^27^ A previous fall can lead to fear of falling and reduced activity.^3^,^28^ Inadequate sleep can also decrease physical performance.^29^ Adequate, restorative sleep helps people physically and mentally with their balance and gait. Not having the recommended amount of sleep has been declared a public health problem by the CDC.^30^ New parents, shift workers, athletes, adolescents, older adults, and approximately 40% of all adults have inadequate sleep.^29^,^31^ However, in this study of West Virginia adults, 83.0% of adults did have adequate sleep.

Chung et al.^31^ found that adults who slept <7.5 hours and older women who slept ≤5 hours had almost a 2-fold higher risk of falls and injuries compared with those who had normal sleep duration. In a cross-sectional study of semi-independent persons in residential care there was an association between risk of falling and nocturnal awakenings (need to urinate, thoughts of generated anxiety or distress, noise, or pain) among adults, aged 65 years and above.^32^ This study found significant associations of falls with not having recommended sleep in adults.

### Strengths and Limitations

This study has several strengths. West Virginia residents, aged ≥45 years were surveyed as representative of the state. As part of Appalachia, the results could be generalized to the 420 counties of the Appalachian Region and 25 million residents as well as other areas with similar rural characteristics. Although West Virginia is unique in many ways, this study supports other research in which falls were related to sleep duration. A validated, nationally representative study of community-dwelling participants was used to extract the data.

The study also has limitations. Its design, as a cross-sectional study, precludes the establishment of cause. Researchers who conducted a population-based study suggest that given the high likelihood of medical conditions, emotional and psychological factors in older adults, it may be difficult to determine whether sleep duration is a major independent factor contributing to falls and injuries,^30^ However, our results indicate sleep duration was an independent factor for falls in adults in West Virginia. Another limitation was insufficient data to determine the relationship of injuries due to falls and sleep duration in this study as there were only 358 reported injuries in the West Virginia BRFSS data.

### Policy Considerations

Sleep disorders have high direct and indirect costs, reduced quality of life, and are common.^33^ Patients, aged ≥45 years, should be counseled to get adequate sleep with the understanding that inadequate sleep is a risk factor for falls.

## IMPLICATIONS

Inadequate sleep duration, based on age, was associated with ≥1 falls within the previous year in a West Virginia Appalachian population.

**SUMMARY BOX**

**What is already known on this topic?** Falls are the third leading cause of fatal unintentional injuries in rural areas.

**What is added by this report?** Sleeping duration should be considered as a preventable action against unintentional fall injuries. People having fall injuries in West Virginia were more likely to have inadequate sleep.

**What are the implications for future research?** Future research should determine whether sleep actually reduces falls in this population. Additionally, research should also be conducted on injuries resulting from falls and the impact of sleep.

## Figures and Tables

**Table 1:** Sample Characteristics, WV BRFSS, 2018 (n=2780)

	Number	Weighted percentage
**Gender**		
Female	1572	51.0
Male	1208	49.0
**Age in years**		
45–55	595	27.6
55–64	853	31.7
65 and above	1332	40.7
**Race/ethnicity**		
White	2624	94.2
Nonwhite	156	5.8
**Education**		
Less than High school	287	15.4
High school	1707	68.0
Some college or above	786	16.6
**Health Insurance**		
Yes	2668	94.5
No	112	5.5
**Body Mass Index**		
Under/normal	711	24.7
Overweight	979	34.3
Obese	1090	41.0
**Exercise**		
Yes	1936	67.3
No	844	32.7
† **Chronic disease**		
Yes	1180	43.5
No	1600	56.5
**Sun exposure of at least 30 minutes in a week**		
Yes	2384	87.0
No	396	13.0
**Hours of sleep per night**		
Less than 6	475	19.3
6 to less than 7	685	24.3
7 to less than 8	779	27.1
8 to less than 9	841	29.3
9 and above	None reported	
¶ **Sleep duration for specific age**		
May be appropriate	2305	80.7
Not recommended	475	19.3
**Fall reported during the year**		
Yes	824	31.1
No	1956	68.9

† chronic disease= positive response to having arthritis, cardiovascular disease, depression, COPD, or diabetes

¶ Based on National Sleep Foundation recommendations where individuals aged 26–64 years, a “not recommended” sleep duration is <6 hours and >10 hours; and for individuals ≥65 years, a “not recommended” sleep duration is <6 hours and >9 hours;^24^ and “may be appropriate” sleep durations in this study were 6 to 10 hours for individuals aged 26–64 years; and 6–9 hours for individuals ≥65 years.^24^

**Table 2:** Bivariate Relationships of Falls and Covariates, WV BRFSS, 2018 (n=2780)

	Number who fell	Weighted Percentage	Number who did not fall	Weighted Percentage	*P-value*
**Gender**					0.9371
Female	474	31.2	1098	68.8	
Male	350	31.0	858	69.0	
**Age in years**					0.0079
45–54	200	34.4	395	65.6	
55–64	262	33.4	591	66.6	
65 and above	362	27.1	970	72.9	
**Race/ethnicity**					0.6658
White	770	31.0	1854	69.0	
Nonwhite	54	33,1	102	66.9	
**Education**					<0.0001
Less than High School	113	43.2	174	56.8	
High School	511	29.7	1196	70.3	
Some college or above	200	25.5	586	74.5	
**Health Insurance**					0.2477
Yes	797	31.4	1871	68.6	
No	27	25.5	85	75.5	
**Body Mass Index**					0.0001
Under/normal	179	27.8	532	72.2	
Overweight	261	27.1	718	72.9	
Obese	384	36.4	706	63.6	
**Exercise**					<0.0001
Yes	511	27.1	1425	72.9	
No	313	39.2	531	60.8	
† **Chronic disease**					<0.0001
Yes	441	39.6	739	60.4	
No	383	24.6	1217	75.4	
**Sun exposure to at least 30 minutes in a week**					0.0006
Yes	669	29.8	1715	70.2	
No	155	40.0	241	60.0	
**Sleep hours**					<0.0001
Less than 6	210	46.1	265	53.9	
6 to less than 7	201	31.5	484	68.5	
7 to less than 8	196	24.8	583	75.2	
8 to less than 9	217	26.7	624	73.3	
¶ **Sleep duration for specific age**					<0.0001
Not recommended	210	46.1	265	53.9	
May be appropriate	614	27.5	1691	72.5	

Percentages may not add to 100% due to missing responses.

† chronic disease= positive response to having arthritis, cardiovascular disease, depression, COPD, or diabetes

¶ Based on National Sleep Foundation recommendations where individuals aged 26–64 years, a “not recommended” sleep duration is <6 hours and >10 hours; and for individuals ≥65 years, a “not recommended” sleep duration is <6 hours and >9 hours;^24^ and “may be appropriate” sleep durations in this study were 6 to 10 hours for individuals aged 26–64 years; and 6–9 hours for individuals ≥65 years.^24^

**Table 3:** Logistic Regression on Falls, WV BRFSS, 2018 (n=2780)

	UOR [95% CI]	*P*-value	AOR [95%CI]	*P*-value
† **Sleep duration for specific age**				
Not recommended	2.26 [1.77, 2.82]	<0.0001	1.77 [1.38, 2.27]	<0.0001
May be appropriate	reference		reference	
**Gender**				
Male			1.07 [0.87, 1.31]	0.5294
Female			reference	
**Age in years**				
45–64			reference	
55–64			0.94 [0.72, 1.24]	0.6733
65 and above			0.66 [0.51, 0.86]	0.0023
**Race**				
White			reference	
Nonwhite			0.89 [0.57, 1.40]	0.6144
**Education**				
Less than High School			1.62 [1.14, 2.29]	0.0066
High School			1.12 [0.89, 1.40]	0.3428
College and above			reference	
**Health Insurance**				
No			0.68 [0.40, 1.14]	0.1382
Yes			reference	
**Body Mass Index**				
Underweight/normal			reference	
Overweight			0.93 [0.71, 1.22]	0.6120
Obese			1.17 [0.91, 1.52]	0.2287
**Exercise**				
No			1.34 [1.08, 1.67]	0.0087
Yes			reference	
¶ **Chronic disease**				
No			reference	
Yes			1.77 [1.44, 2.18]	<0.0001
**Sun exposure to at least 30 minutes in a week**				
No			1.30 [0.97, 1.73]	0.0790
Yes			reference	

† Based on National Sleep Foundation recommendations where individuals aged 26–64 years, a “not recommended” sleep duration is <6 hours and >10 hours; and for individuals ≥65 years, a “not recommended” sleep duration is <6 hours and >9 hours;^24^ and “may be appropriate” sleep durations in this study were 6 to 10 hours for individuals aged 26–64 years; and 6–9 hours for individuals ≥65 years.^24^

¶ chronic disease=positive response to having arthritis, cardiovascular disease, depression, COPD, or diabetes
